# Lipid Nanoparticle (LNP) ‐A Vector Suitable for Evolving Therapies for Advanced Hepatocellular Carcinoma (HCC)

**DOI:** 10.1002/gch2.202400217

**Published:** 2024-11-25

**Authors:** Mingxuan Zhang, Ruiping Guo, Zhuhui Yuan, Hao Wang

**Affiliations:** ^1^ Department of Radiation Oncology Cancer Center of Peking University Third Hospital Peking University Third Hospital Haidian, 49 Huayuan North Road Beijing 100191 China

**Keywords:** chemotherapies, gene therapies, hepatocellular carcinoma, immune therapy, lipid nanoparticles

## Abstract

Hepatocellular carcinoma (HCC) stands as the predominant form of primary liver cancer, characterized by a dismal prognosis. Therapeutic options for advanced HCC remain sparse, with efficacy significantly hampered by the emergence of drug resistance. In parallel with research into novel pharmacological agents, advances in drug delivery systems represent a promising avenue for overcoming resistance. Lipid nanoparticles (LNPs) have demonstrated considerable efficacy in the delivery of nucleic acid‐based therapeutics and hold potential for broader applications in drug delivery. This review describes the development of LNPs tailored for HCC treatment and consolidates recent investigations using LNPs to target HCC.

## Graphical abstract

This review focuses on the challenges in the treatment of hepatocellular carcinoma (HCC) and how lipid nanoparticles (LNPs) have demonstrated considerable efficacy in the delivery of nucleic acid‐based therapeutics and have shown potential in chemotherapy, immunotherapy, and gene therapy. The study summarizes the evolution of the use of LNPs to target HCC and suggests that LNPs are a future trend for HCC treatment.

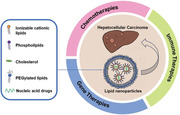

## 1 Introduction

Liver cancer ranks as the third leading cause of cancer‐related mortality and the eighth most prevalent malignancy globally.^[^ ^1^ ^]^ Hepatocellular carcinoma (HCC) constitutes ≈90% of all primary liver cancer cases.^[^ ^2^ ^]^ Recent advancements in HCC treatment encompass surgical resection, liver transplantation, image‐guided tumor ablation, and image‐guided transcatheter tumor therapy, all of which have demonstrated efficacy.^[^ ^3^ ^]^ However, the majority of HCC patients present with advanced disease at diagnosis, with only 15% qualifying for potentially curative surgical interventions. Moreover, recurrence rates post‐resection are alarmingly high, reaching up to 70% within five years.^[^ ^4^ ^]^ Therapeutic options remain limited for patients with advanced‐stage HCC.^[^ ^5^ ^]^ While systemic therapies and immunotherapy can extend survival, resistance to these treatments is prevalent, rendering the prognosis for HCC particularly bleak despite substantial progress in immunotherapy.

The quest for new therapeutic strategies for advanced HCC is intrinsically linked to developing targeted therapies. With the advent of gene therapy, nucleic acid‐based drugs have garnered significant attention. LNPs, having undergone extensive research, represent the most advanced non‐viral gene delivery systems and are less immunogenic than viral vectors.^[^ ^6^ ^]^ In 2013, RNA interference (RNAi) therapy using anti‐vascular endothelial growth factor (anti‐VEGF) and anti‐Kinesin spindle proteins (anti‐KSP) siRNAs delivered by LNPs was first evaluated in HCC patients. The investigational drug ALN‐VSP administered intravenously, was safe and well‐tolerated, demonstrating efficacy through observable anti‐VEGF and anti‐KSP effects in patients.^[^ ^7^ ^]^ Numerous RNAi targets have been identified, influencing HCC therapy and augmenting other therapeutic modalities, offering new possibilities for HCC treatment.

**Figure 1:**
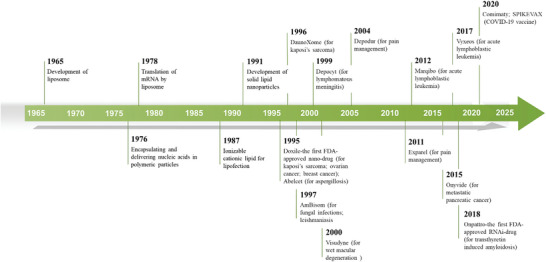
The timeline of the development of liposomes/LNPs.

Additionally, LNPs have been employed for mRNA delivery in cancer immunotherapy as tumor vaccines or for expressing desired proteins in cells.^[^ ^8^, ^9^ ^]^ Approving the COVID‐19 mRNA‐LNP vaccine has significantly heightened interest in LNPs, positioning them as the preferred choice for nucleic acid drug delivery.^[^ ^10^ ^]^ **Figure** **1** shows the timeline of the development of liposomes/LNPs, and **Table** **1** shows progress made in LNP‐based therapies for HCC This review synthesizes the advancements and applications of LNPs in HCC therapy.

**Table 1:** Representative clincal trials of LNP‐based therapies for HCC.

Name	Targeted gene	ClinicalTrials.gov identifier	Phase
DCR‐MYC	MYC	NCT02314052	I/II
WGI‐0301	Akt‐1	NCT06309485	II
OTX‐2002	MYC	NCT05497453	I/II

## 2 Challenges in the Treatment of HCC

### 2.1 Pathologic Changes in HCC Affect Drug Distribution

The poor accumulation of therapeutic agents in HCC following systemic administration is a critical factor contributing to drug resistance. Major risk factors for HCC, including viral infections, alcoholic liver disease, and non‐alcoholic fatty liver disease (NAFLD), often lead to chronic liver injury that progresses from fibrosis to carcinoma. Liver fibrosis, present in ≈90% of HCC cases, results in numerous pathological changes that significantly impede drug delivery.^[^ ^11^ ^]^

Chronic inflammatory injury and repair within the liver create a vicious cycle in which hepatic stellate cells (HSCs) play a critical role by mediating the accumulation of extracellular matrix (ECM) proteins. This leads to the replacement of collagen IV by fibrillar collagens I and III in the perisinusoidal space. This accumulation and pro‐inflammatory factors mark the early stages of chronic liver injury, culminating in the capillarization of liver sinusoidal endothelial cells (LSECs) and promoting fibrosis progression.^[^ ^12^, ^13^ ^]^ The capillarization of LSECs implies the disappearance of the hepatic sinusoids' endothelial window; the double barrier formed by LSECs and ECM deposited in the perisinusoidal space will make it difficult for drugs to pass through.^[^ ^14^ ^]^ Meanwhile, portal hypertension and HCC are highly comorbid, and viral infection‐induced hepatic fibrosis can also induce early portal hypertension, which results in the blood supply to cancerous foci being mainly from the hepatic artery rather than the portal vein supplying the normal liver, making it difficult to reach for intravenous drug delivery.^[^ ^14^, ^15^ ^]^

### 2.2 Immune Microenvironment‐Mediated Drug Resistance in HCC

During the pathogenesis of HCC, which evolves from chronic inflammation to fibrosis and carcinoma, the immune microenvironment shifts toward immunosuppression. This transformation is closely linked to the low responsiveness of HCC to systemic therapy.^[^ ^16^ ^]^ Notably, the severity of liver fibrosis correlates positively with the expression of immune checkpoint molecules, indicating T cell depletion and enhanced immune escape within fibrotic liver tissues.^[^ ^17^, ^18^ ^]^ GOLM1 contributes to the pathogenic progression of HCC by up‐regulating PD‐L1 expression, thereby facilitating immune escape.^[^ ^17^ ^]^ HSCs within the fibrotic microenvironment further influence the tumor microenvironment (TME), as they release multiple cytokines that promote M2 macrophage polarization.^[^ ^19^ ^]^

Epidemiologic studies have indicated a rising incidence of NAFLD‐related HCC.^[^ ^20^ ^]^ Notably, HCC associated with NAFLD is less responsive to immunotherapy than HCC driven by HBV infection, the most prevalent risk factor.^[^ ^21^ ^]^ Research into anti‐immunotherapy mechanisms in NAFLD emphasizes alterations in T cells within the immune microenvironment. In NASH mice, CD8+ T cells with inflammatory phenotypes exhibit abnormal activation and reduced mobility, impairing the efficacy of immune checkpoint inhibitors (ICI).^[^ ^22^, ^23^ ^]^ Patients with NAFLD‐HCC treated with PD‐1 inhibitors show increased CD8 + PD1+ T cells, exacerbating T cell exhaustion.^[^ ^21^ ^]^ These findings suggest that CD8+ T cells may act as a negative factor in the TME of NAFLD‐related HCC. Additionally, lipid dysmetabolism plays a critical role in the pathogenesis of NAFLD‐related HCC. The accumulation of free fatty acids, particularly linoleic acid, leads to reactive oxygen species (ROS)‐mediated oxidative damage via mitochondrial disruption, resulting in the loss of CD4+ T cells.^[^ ^24^, ^25^ ^]^

The TME is a common feature in the pathogenesis of HCC induced by various risk factors, acting as a predisposing factor for HCC and a significant barrier to its treatment. The intricate interactions within the TME complicate the resolution of immunotherapy resistance by targeting a single molecule. Therefore, simultaneously targeting multiple components and disrupting the network within the TME presents a promising strategy to overcome immunotherapy resistance in HCC.

### 2.3 Involvement of lncRNAs in the Drug Resistance of HCC

Long non‐coding RNAs (lncRNAs) arenon‐coding RNA cells more than 200 nt in length, which are important components of signaling networks and targets of drug resistance in cancer. An important downstream signaling molecule of lncRNAs is miRNAs, 20–25 nt in length, which specifically inhibit post‐transcriptional expression of genes, while its upstream lncRNAs regulate the gene silencing effect of miRNAs by preventing their binding of miRNAs to their target mRNAs regulates the gene silencing effect of miRNAs.^[^ ^26^, ^27^ ^]^ Therefore, the LncRNA‐miRNA network is an important regulatory system for cellular life activities, including its drug resistance. In addition, LncRNAs are involved in regulating a variety of signaling pathways known to be associated with drug resistance, including PI3K/AKT, Wnt/β‐catenin, MAPK, and PTEN signaling pathways.^[^ ^28^ ^]^ Table 1 lists the lncRNAs and corresponding signaling pathways and mechanisms associated with HCC drug resistance.

## 3 Progress in LNPs as a Carrier for Targeting HCC

### 3.1 Why Choose LNPs as a Carrier for Targeting HCC

Table 3 shows viral and non‐viral methods for transfering nuclear‐based drugs. Although viral vectors have advantages in transfection efficiency, its drawbacks are equally obvious higher immunogenicity. Drawbacks in safety and capacity make them slightly inferior to LNPs which have higher liver specificity, better safety, and higher capacity. Polymer nanoparticles, on the other hand, are characterized by poor targeting, high toxicity, and are even far less discussed than LNPs. The latest delivery of CRISPR‐Cas9 gene editing system NTLA‐2002 in the form of mRNA via LNPs has already been approved by the FDA for human clinical trials. LNPs have been at the forefront of gene therapy.

### 3.2 Composition and Characterization of LNPs

#### 3.2.1 BASIC Structure of LNPs

The typical structure of LNPs comprises four essential components: ionizable cationic lipids, phospholipids, cholesterol, and PEGylated lipids.^[^ ^9^, ^64^ ^]^ These components confer distinct advantages to LNPs in delivering nucleic acid drugs. The nucleic acid component of these drugs is negatively charged, while the ionizable cationic lipids, a critical component of LNPs, feature a positively charged hydrophilic amino head in acidic environments. This allows for the encapsulation of nucleic acids within the lipid nanoparticles via electrostatic interactions. In a neutral plasma environment, these lipids are uncharged, thus avoiding uptake by monocyte macrophages, reducing toxicity, and finally becoming endosomes for release into the cytoplasm through megacellular drinking.^[^ ^65^ ^]^ Among them, the key to improve the delivery efficiency is the release of LNPs from endosomes, i.e., the efficiency of endosome escape. The mechanism of endosomal escape is closely related to ionizable cationic lipids. One theory hypothesizes that cationic lipids interact with anionic phospholipids in the endosomal membrane to form a non‐bilayer structure and disrupt the endosomal membrane, thereby releasing nucleic acids.^[^ ^66^ ^]^ In contrast, another hypothesis suggests that the delivery efficiency of cationic lipids can be attributed to the proton sponge phenomenon, in which buffering cationic polymers bind protons in the endosomes and activate the endosomal proton pump, allowing chloride ions to enter the endosomes along with the protons, elevating the osmotic pressure and causing the endosomes to swell and rupture.^[^ ^67^ ^]^ However, the inefficiency of endosomal escape and the unidentified mechanism still make it a rate‐limiting step in the delivery of LNPs.^[^ ^68^ ^]^ PEGylated lipids prevent the aggregation of LNPs, while phospholipids contribute to nanoparticle formation. Cholesterol enhances the stability of nucleic acid‐lipid nanoparticles and promotes their fusion with cellular membranes.^[^ ^9^, ^69^, ^70^ ^]^

Based on this basic structure, the newly developed LNPs have a better ratio of lipid components, or a more appropriate N: P. Modifications can be introduced to endow LNPs with enhanced biological functions, thereby maximizing their functional flexibility. Optimizing the lipid composition, such as altering the structure of ionizable cationic lipids to modify their apparent pKa, represents a promising direction for enhancing LNP performance. The functionality of LNPs is significantly influenced by their apparent pKa, highlighting the importance of this parameter in the design and optimization of these delivery systems.^[^ ^71^ ^]^

#### 3.2.2 LNPs Characterization for Targeting

One of the compelling reasons for the promise of LNPs in targeted therapies for HCC is their ability to achieve passive targeting. The hepatic sinusoids, characterized by their discontinuous vasculature and slow blood flow, facilitate the natural accumulation of LNPs without additional modifications.^[^ ^72^, ^73^, ^74^ ^]^ Moreover, the enhanced permeability and retention (EPR) effect has long been recognized for its role in nanoparticle (NP) accumulation in tumors. This phenomenon allows NPs to extravasate through the highly permeable blood vessels surrounding tumors, which are more accessible to macromolecules. Furthermore, the reduction in lymphatic clearance enhances NP retention in the tumor environment, a process influenced by the physicochemical properties such as the size of the NPs.^[^ ^75^, ^76^, ^77^ ^]^ However, despite the significant impact of the EPR effect observed in animal experiments, clinical evidence supporting its efficacy remains elusive.^[^ ^78^ ^]^

#### 3.2.3 Immunogenicity of LNPs

LNPs not only elicit immune responses, but the nature of these responses is contingent upon the composition of the LNPs. Researchers have concentrated on the immunogenic potential of LNPs, particularly in the context of RNA vaccines. Chen et al.^[^ ^79^ ^]^ reviewed evidence demonstrating that all four primary components of LNPs can trigger immune responses, leading to inflammation. Following the approval of COVID‐19 mRNA‐LNP vaccines, numerous adverse reactions have been documented, with PEG‐lipid‐mediated IgE‐associated allergic responses and IgM complement pathway activation identified as key mechanisms.^[^ ^80^ ^]^

Phosphatidylserine (PS), a component known to influence immunomodulation, has been shown to induce immune tolerance when incorporated into LNPs, primarily through the expansion of regulatory T cells (Tregs).^[^ ^79^, ^81^, ^82^ ^]^ Substituting cholesterol with its analog, β‐sitosterol can inhibit or promote T cell proliferation.^[^ ^79^, ^83^ ^]^ Since inflammatory microenvironments foster tumor growth, replacing traditional LNP components with those with anti‐inflammatory properties may offer therapeutic advantages in oncology. However, the immunogenicity and immune tolerance induced by different LNP components and their underlying mechanisms remain underexplored in the context of HCC‐targeted therapies. Furthermore, the presence of anti‐PEG IgE and IgM in humans suggests the potential for similar adverse effects with future LNP‐based antitumor drugs as seen with mRNA‐LNP vaccines. This represents a critical area for further research and improvement.

### 3.3 Optimization of LNP Targeting HCC

#### 3.3.1 Enhancement of LNP Targeting Ability and Transfection Rate

Akinc et al.^[^ ^84^ ^]^ discovered that ionizable LNPs (iLNPs) adsorbing apolipoprotein E (ApoE) can enhance the uptake of LNPs via the low‐density lipoprotein receptor (LDL‐R) pathway, thereby targeting hepatocytes endogenously. Building on this finding, Johnson's team screened a chemically diverse library of ionizable cationic lipids and identified the amino lipid 5A2‐SC8.^[^ ^85^ ^]^ LNPs formulated with 5A2‐SC8/DSPC/Chol/PEG‐DMG exhibited higher in vivo delivery efficiency compared to another construct with similar physical properties, 3A5‐SC14/DSPC/Chol/PEG‐DMG. This discrepancy arises because different LNPs adsorb distinct proteins, forming varied protein coronas during delivery. Specifically, 5A2‐SC8 LNPs adsorb a significant amount of ApoE, facilitating hepatocyte endocytosis via LDL‐R, whereas 3A5‐SC14 LNPs predominantly adsorb albumin, which is readily phagocytosed by Kupffer cells. Notably, 3A5‐SC14 LNPs demonstrated a higher hepatocyte accumulation rate in vitro than 5A2‐SC8. Further studies on LNP protein coronas revealed that altering the carbon chain length of PEG lipids and the molar percentage of PEGs also affects the adsorbed protein corona. Pre‐incubation of ApoE with various LNPs indicated that ApoE promoted uptake only in C14‐PEG LNPs, not C18‐PEG LNPs, highlighting that small differences in LNP composition can influence both the type of protein corona and its effect on LNP uptake.^[^ ^86^, ^87^ ^]^

The PEG shield significantly impacts LNP uptake rates in cells and can cause substantial disparities between in vitro and in vivo results. Using LNPs composed of PEG molecules with different lipid anchors to target TetR‐ODC‐Luc cells and an in vivo tumor model, it was observed that LNPs containing PEG‐DPPE and PEG‐DSPE exhibited low uptake rates in vitro but successfully delivered siRNAs to tumors in vivo, whereas LNPs containing PEG‐DMPE showed the opposite results.^[^ ^88^ ^]^ This underscores the importance of selecting appropriate PEG modifications in LNP construction.

Although viral vectors offer superior transfection efficiency compared to LNPs, LNPs provide greater safety and loading capacity.^[^ ^89^ ^]^ Phosphatidylserine (PS) is a crucial cofactor for many viral transfections. Lotter et al.^[^ ^90^ ^]^ developed PS‐modified LNPs to target Huh7 cells, significantly increasing the transfection rate. The optimal PS percentage to enhance transfection was determined to be 2.5–5%.

LNPs undergo several stages from preparation to delivery: loading, circulation in the bloodstream, cellular uptake, and cytoplasmic release. Second‐generation ionizable cationic lipid LNPs are designed to be positively charged in the acidic environment of endosomes, facilitating cargo release. Zhao et al.^[^ ^91^ ^]^ developed a novel sTPssOLP LNP, incorporating disulfide‐modified PEG lipids with transferrin (Tf) insertion. This modification prolongs the LNPs' circulation time in vivo, increasing accumulation in HCC. The disulfide bonds are reduced in the cytoplasm, enabling precise cargo release. The efficacy of sTPssOLP as a new carrier was demonstrated in mouse tumor‐bearing experiments.

#### 3.3.2 Insertion of Drug Molecules into the Lipid Bilayer of LNP to Achieve Co‐Delivery

Chemotherapy or immunotherapy alone often fails to achieve the desired therapeutic outcomes in hepatocellular carcinoma (HCC) due to mechanisms of drug resistance and immune evasion. Consequently, combination drug therapy has emerged as a promising strategy to overcome these challenges. By co‐delivering therapeutic agents and auxiliary drugs with sensitizing effects, synchronous drug distribution can be achieved, resulting in a synergistic effect that enhances tumor eradication. The first choice for LNP co‐delivery is still nucleic acids, while Ball et al.^[^ ^92^ ^]^ explored the co‐delivery of siRNA and mRNA and found that co‐delivery of both nucleic acids could enhance the transfection efficiency of LNPs, and the use of negatively charged polymer polystyrene sulfonate (PSS) to replace co‐delivered RNAs resulted in an increase in the efficiency of single RNA delivery. When considering co‐delivery, RNA drugs with synergistic effects can be prioritized, and the selection of suitable co‐delivery drugs is also a way to improve the co‐delivery efficiency. Botanical drugs frequently serve as auxiliary agents, with substantial experimental evidence supporting their efficacy in combination with various chemotherapeutic agents, including 5‐fluorouracil (5‐Fu), sorafenib, and cisplatin.^[^ ^93^ ^]^

However, the simultaneous encapsulation of two drugs presents issues such as uncontrollable encapsulation ratios and low drug loading efficiency.^[^ ^93^ ^]^ Xu et al.^[^ ^94^ ^]^ developed glycyrrhetinic acid‐LNPs (GLLNPs) to address these challenges. Glycyrrhetinic acid (GL), which possesses a cholesterol‐like structure, plays a role in remodeling the immune microenvironment, inhibiting M2 macrophage polarization, and modulating immunosuppressive cells.^[^ ^95^, ^96^ ^]^ By substituting cholesterol with GL in the LNPs and incorporating triptolide (TP) into the lipid bilayer, GLLNPs enable the co‐delivery of GL and TP. This innovative approach suggests that identifying drug molecules with both pharmacodynamic effects and the potential to form lipid backbones could represent a new direction for effective co‐delivery systems. Rong et al.^[^ ^97^ ^]^ added DLin‐MC3‐DMA (MC3) in addition to the traditional LNPs components when co‐delivering camptothecin and MiR‐145, and MC3 not only wrapped the nucleic acids individually within LNPs but also facilitated the endosomal escape of LNPs and enhanced their transfection efficiency.

#### 3.3.3 Altered Adaptation of LNPs to Transport Non‐Nucleic Drugs

LNPs exhibit positive charges in acidic environments, facilitating the encapsulation of negatively charged nucleic acids. However, co‐delivering proteins with nucleic acids requires additional strategies, such as adding negatively charged modifications to ensure efficient transport, albeit often at suboptimal concentrations. Haley et al.^[^ ^98^ ^]^ addressed this challenge by screening an ionizable lipid library of structural analogs to C12‐200, ultimately developing the formulation C14‐4/DOTAP/DOPE/Chol/PEG. This formulation was designed to deliver the negatively charged modified RAS inhibitor K27‐D30. The protein was successfully delivered, significantly reducing tumor burden in a mouse model of hydrodynamic tail vein injection (HTVI)‐induced HCC.

## 4 Developing LNPs Targeting HCC

### 4.1 LNPs for Chemotherapies

Chemotherapy plays a crucial role in the treatment of advanced HCC. To date, the multi‐targeted receptor tyrosine kinase (TKI) inhibitor sorafenib and other TKI inhibitors continue to serve as the first‐line therapeutic regimen for systemic chemotherapy in HCC.^[^ ^99^ ^]^ Although LNPs are not typically the primary choice for delivering non‐nucleic acid drugs, they can effectively transport negatively charged modified proteins through classical LNP formulations.^[^ ^98^ ^]^ Solid lipid nanoparticles (SLNs), on the other hand, are more prevalently used for encapsulating non‐nucleic acid drugs. SLNs exhibit hepatic enrichment akin to LNPs and can release their drug payload within the endosome post‐endocytosis.^[^ ^100^ ^]^ Furthermore, unlike LNPs, SLNs offer advantages in encapsulating hydrophobic and hydrophilic drugs, extending their applications to delivering non‐nucleic acid therapeutics such as natural compounds with anti‐cancer properties.^[^ ^100^ ^]^ (**Table** **2**).

**Table 2:** LncRNAs related to HCC drug resistance.

LncRNA	Relevant signaling pathways or mechanisms	Drugs	References
LINC01056	LINC01056/PPARα/FAO	Sorafenib	[29]
NEAT1	NEAT1/miR‐149‐5p/AKT1	Sorafenib	[30]
HCG18	HCG18/miR‐450b‐5p/GPX4; Ferroptosis	Sorafenib	[31]
HEIH	HEIH/miR‐98‐5p‐ PI3K/AKT	Sorafenib	[32]
TTN‐AS1	TTN‐AS1/miR‐16‐5p/cyclin E1; PTEN/Akt pathway	Sorafenib	[33]
PTOV1‐AS1	PTOV1‐AS1/miR‐505	Sorafenib	[34]
URB1‐AS1	Ferroptosis	Sorafenib	[35]
LIMT	LIMT/miR‐665	Sorafenib	[36]
BBOX1‐AS1	BBOX1‐AS1/miR‐361‐3p/PHF8	Sorafenib	[37]
AC026401.3	AC026401.3//OCT1/E2F2	Sorafenib; Lenvatinib	[38]
SNHG3	SNHG3/miR‐128/CD151	Sorafenib	[39]
TRERNA1	TRERNA1/miR‐22‐3p/NRAS	Sorafenib	[40]
hMTR4	hMTR4/PDIA3P1/miR‐125/124/TRAF6; NF‐κB pathway	Adriamycin	[41]
MALAT1	MALAT1/miR‐3129‐5p/Nova1	Adriamycin	[42]
HANR	HANR/GSKIP/GSK3β	Doxorubicin	[43]
SNHG16	SNHG16/hsa‐miR‐93	5‐fluorouracil	[44]
KRAL	KRAL/miR‐141/Keap1	5‐fluorouracil	[45]
LINC02362	LINC02362/hsa‐miR‐18a‐5p/FDX1	Oxaliplatin	[46]
PCGEM1	PCGEM1/miR‐129‐5p/ETV1	Oxaliplatin	[47]
DUBR	SP1/DUBR/E2F1/CIP2A;Notch1 pathway	Oxaliplatin	[48]
NR2F1‐AS1	NR2F1‐AS1/miR‐363/ABCC1	Oxaliplatin	[49]
LINC01134	LINC01134/SP1/p62	Oxaliplatin	[50]
ZEB2‐19	ZEB2‐19/TRA2A/RSPH14	Lenvatinib	[51]
HOTAIR(M1)	HOTAIRM1/miR‐34a/Beclin‐1	Lenvatinib	[52]
	HOTAIR/miR‐34a; Akt pathway	Paclitaxel	[53]
	HOTAIR/miR‐217	Sorafenib	[54]
DUXAP8	DUXAP8/SLC7A11; Ferroptosis	Sorafenib	[55]
	DUXAP8/miR‐485‐5p/FOXM1	Olaparib	[56]

### 4.2 LNPs for Immune Therapies

Emerging evidence indicates that the TME significantly contributes to therapeutic resistance and facilitates tumor progression.^[^ ^106^, ^107^ ^]^ Within the TME, tumor cells frequently communicate with their surrounding microenvironment through various mediators, attracting and reprogramming immune cells to become components of the suppressive milieu.^[^ ^108^, ^109^ ^]^ The expression of exogenous mRNAs within the TME can effectively modulate immune cells and remodel the immune microenvironment. Lipid nanoparticles (LNPs) are the preferred vehicle for delivering mRNA therapeutics, and the FDA has already approved two mRNA‐LNP formulations (**Table** **3**).

**Table 3:** Viral and non‐viral methods summarize.

	Vector	Target tissues or cells	Capacity/size	Transfection efficiency	References
Viral methods	Adeno‐associated virus (AAV)	liver, striated muscles, and the CNS	Under 5.0 kb	Seropositive for antibodies against AAV significantly reduces the transfection efficiency.	[57]
	Adenovirus (AdV)	Cancer cells	8–37 kb	Efficient but transient	[58, 59]
	Lentivirus (LV)	CD34+ hematopoietic stem cell, T cells (ex vivo)	9–10 kb	Efficient and stable	[60, 61]
Non‐viral methods	Lipid nanoparticles (LNP)	liver	20–200 nm, affected by N/P ratio and size of nuclear cargo	Lower than viral vectors	[62]
	Polymer nanoparticles	Low specificity	10–100 nm	Lower than viral vectors	[63]

### 4.3 LNPs for Gene Therapies

Gene therapy represents a novel therapeutic approach whereby therapeutic nucleic acids are introduced into the human body in vitro or in vivo to achieve therapeutic outcomes. In vitro therapies involve extracting cells from the patient's body, reprogramming specific cells outside the body, and subsequent re‐infusion of these cells into the patient. Chimeric antigen receptor T‐cell (CAR‐T) therapy is the most widely utilized in vitro gene therapy and is currently the only FDA‐approved gene therapy.^[^ ^115^ ^]^ In contrast, in vivo therapies, which entail the direct delivery of therapeutic nucleic acids to the patient, are increasingly being researched to treat HCC. The in vivo delivery of nucleic acids mediated by LNPs has been extensively explored, with significant advances in RNAi targeting^[^ ^116^ ^]^ (**Table** **4**).

**Table 4:** LNPs for Chemotherapies.

Experimental type	Carrier components	Cargo	Results	References
In vitro and in vivo	C14‐4, DOTAP, DOPE, cholesterol, lipid‐anchored PEG polymer	K27‐D30	These LNPs deliver K27‐D30 to the cytosol of cancerous cells in the liver, inhibiting RAS‐driven growth and ultimately reducing tumor load in an HTVI‐induced mouse model of hepatocellular carcinoma.	[98]
In vitro and in vivo	DODMA, cholesterol, PEG bad egg	Valeric acid	Lipid‐based nanoparticle‐encapsulated valeric acid significantly reduces the tumor burden and improves survival.	[101]
In vitro and in vivo	HSPC, DSPE‐PEG2000	CU1	In vitro, the uptake efficiency of CU1‐LSLN to MHCC‐97H cells was significantly higher than that of CU and CU1, and the expression levels of NF‐κB, COX‐2, MMP‐2, MMP‐9, and uPA decreased. In vivo, CU1‐LSLN prolonged the retention time of the drug.	[102]
In vitro and in vivo	HSPC, PVPK15	CU and PTX	In vitro, PTX of CU‐PTX‐LNP showed a decrease in the IC50 compared to free PTX. In vivo, CU‐PTX‐LNP displayed excellent biosafety, significant anti‐tumor benefits, and enhanced pharmacokinetic behavior with longer mean residence time and half‐life relative to free drugs.	[103]
In vitro and in vivo	capmul MCMC10, stearic acid, cetyl alcohol, GMS, Compritol ATO888, soy lecithin	Resveratrol (RV)	In vitro, RV‐c‐SLN has the lowest IC50 on HepG2 compared to the RV solution. In vivo, RV‐c‐SLN showed a significant reduction of tumor volume and higher accumulation in the tumor tissue over RV solution and RV‐SLN.	[104]
In vitro and in vivo	TM, ePC, Gal‐DOPE (for active targeting)	Docetaxel	Cytotoxicity of tSLNs superior to Taxotere and non‐targeted SLNs (nSLNs). Better uptake by hepatoma cells was why tSLNs showed better tolerance and antitumor efficacy in murine model‐bearing hepatoma compared with Taxotere or nSLNs.	[105]

## 5 Conclusion

The current direction of cancer treatment development increasingly emphasizes enhancing precision targeting to minimize collateral damage to normal tissues and improve quality of life during therapy. For patients with advanced HCC, targeted therapy represents a promising avenue. Due to the pathophysiological characteristics of HCC, intravenously administered drugs tend to accumulate in the liver preferentially. LNPs emerge as promising targeting vectors that can be easily functionalized by incorporating different molecules and adjusting the lipid composition. The precise targeting capabilities of LNPs enhance drug efficacy, reduce toxicity, and consequently improve the safety profile of therapeutics. The versatility of LNPs is further demonstrated when combined with other therapies, such as immune checkpoint inhibitors, to potentiate the therapeutic effects of existing treatments, as validated in experimental studies. The successful deployment of the COVID‐19 mRNA vaccine underscores the feasibility of mass‐producing LNPs and their application in real‐world treatments. This review delineates the challenges in systemic drug delivery for HCC, highlights the research advancements in LNP technology, and elucidates their applications and advantages.(**Tables** **5** and **6**).

**Table 5:** LNPs for immune therapies.

Experimental type	Carrier components	Cargo	Result	References
In vitro and in vivo	PPZ‐A10, DOPE, cholesterol and DMG‐PEG2000	GPC3‐specific CAR mRNA	In vitro, PPZ‐A10 LNPs preferentially delivered mRNA to macrophages rather than tumor cells. In vivo, mice treated with LNPs generating CAR‐Ms significantly elevate the phagocytic function of liver macrophages, reduce tumor burden, and increase survival time in an HCC mouse model.	[110]
In vitro and in vivo	Not mentioned	Modified IL‐12 mRNA	Treatment with IL‐12‐LNP significantly reduced liver tumor burden in HCC transgenic mice. IL‐12‐LNP elicited marked infiltration of activated CD44+ CD3+ CD4+ T helper cells into the tumor and increased the production of Interferon γ (IFNγ).	[111]
In vitro and in vivo	DLin‐MC3‐DMA, DSPC, cholesterol, DSPE‐PEG2000	OX40L mRNA	In vivo, OX40L acts as a costimulator, significantly increasing CD4+ and CD8+ T cells. OX40L mRNA‐LNPs reduced tumor growth and increased the survival of mice bearing H22 tumors.	[112]
In vitro and in vivo	DOTAP	tumor‐derived RNA from Hepa1‐6 cells	RNA LNP vaccines could efficiently promote DC maturation and induce specific CTL against Hepa1‐6 cells in vitro. Preventive immunized mice with RNA LNPs can effectively prevent and inhibit the growth of Hepa1‐6 tumors.	[113]
In vitro and in vivo	DLin‐MC3‐DMA, DOPE, cholesterol, C14‐PEG2000	BisCCL2/5i mRNA or PD‐Li mRNA	BisCCL2/5i significantly induces the polarization of TAMs toward the antitumoral M1 phenotype and reduces immunosuppression in the tumor microenvironment. The combination of BisCCL2/5i with PD‐Li achieves long‐term survival in mouse models of primary liver cancer.	[114]

**Table 6:** LNPs for gene therapy.

Experimental type	Carrier components	Cargo	Result	References
In vitro and in vivo	cationic lipid RL01, DSPC, cholesterol, and DMPE‐PEG2000	anti‐miR‐17 family oligonucleotides	In vitro, the LNP‐mediated delivery of anti‐miR‐17(5) derepressed miR‐17 family targets in orthotopic Hep3B tumors. In vivo, the LNP dose‐dependently derepressed miR‐17 family targets the tumor tissues and suppresses tumor growth. Mechanically speaking, anti‐miR‐17 therapy induces apoptosis and cell cycle arrest by specifically de‐repressing several targets in the MYC pathway, thus delaying tumorigenesis in MYC‐driven HCCs.	[117, 118]
In vitro and in vivo	DL‐103, DSPC, cholesterol, DSG‐PEG2K	Dicer‐substrate siRNA (DsiRNA) targeting CTNNB1	DsiRNA delivery was homogeneous in tumor sections, selective over normal liver, and independent of apolipoprotein‐E binding. Significant tumor growth inhibition was achieved in Wnt‐dependent colorectal and hepatocellular carcinoma models but not in Wnt‐independent tumors.	[119]
In vitro and in vivo	Lipids for selection	siRNAs for selection	After 2 steps for selection, A‐066/TMH400/DSPC/cholesterol was the most active LNP in vivo. In the selection of therapeutic targets and siRNA molecules, targeting CDCA1_1.2 significantly inhibited the growth of the orthotopic HuH7 liver tumor without significant cytokine/chemokine induction. A‐066/TMH400‐CDCA1_1.2 was a promising therapy for HCC.	[51]
In vivo	3‐((5‐(dimethylamino) pentanoyl)oxy) −2,2‐bis (((3‐pentyloctanoyl)oxy)methyl)propyl 3‐pentyloctanoate, DPPC, cholesterol, GS‐020	KNTC2 siRNAs	KNTC2 siRNA‐LNP significantly inhibited the in vivo growth of Hep3B. KNTC2 siRNAs‐LNP led to tumor shrinkage and protected normal hepatocytes, decreasing plasma AST and ALT levels.	[120]
In vitro and in vivo	DODMA, eggPC, cholesterol, PEG‐lipid	miR‐122	In vitro, LNP‐DP1‐mediated transfection of a miR‐122 mimic to HCC cells down‐regulated miR‐122 target genes by >95%. In vivo, injecting LNP‐DP1/miR‐122 intratumorally to HCC models could significantly suppress angiogenesis in tumors and lead to suppression of tumor growth.	[121]
In vitro	DLin‐MC3‐DMA, cholesterol, DSPC, DMG‐PEG2000	CD47_siRNA	Values of CD47 knockdown efficiency were above 90% in HepG2, and the higher the knockdown of CD47 in cancer cells, the higher the percentage of apoptosis. However, unlike small‐GO‐PEG‐PAMAM/CD47_siRNA, CRT levels were not correlated with cancer apoptosis.	[122]
In vitro and in vivo	mPEG‐PLA, BHEM‐Chol	YTHDF1 siRNA	YTHDF1 promotes NASH‐HCC tumorigenesis via EZH2‐IL‐6 signaling, which recruits and activates MDSCs to cause cytotoxic CD8+ T‐cell dysfunction. LNP‐siYthdf1 markedly decreased YTHDF1 protein levels in mouse NASH‐HCC tumors. LNP‐siYthdf1 combined with anti‐PD‐1 synergistically decreased tumor burden and cell proliferation, which means LNP‐siYthdf1 has potential in anti‐PD‐1 therapy in NASH‐HCC.	[123]

## Conflict of Interest

The authors declare no conflict of interest.
